# A Population-Based Study of the Prevalence and Correlates of Self-Harm in Juvenile Detention

**DOI:** 10.1371/journal.pone.0146918

**Published:** 2016-01-13

**Authors:** Hygiea Casiano, Shay-Lee Bolton, Keith Hildahl, Laurence Y. Katz, James Bolton, Jitender Sareen

**Affiliations:** 1 Department of Psychiatry, University of Manitoba, Winnipeg, Manitoba, Canada; 2 Departments of Community Health Sciences and Psychiatry, University of Manitoba, Winnipeg, Manitoba, Canada; 3 Departments of Psychiatry, Psychology, and Community Health Sciences, University of Manitoba, Winnipeg, Manitoba, Canada; Medical University of Vienna, AUSTRIA

## Abstract

**Background:**

Suicide is the number one cause of death among incarcerated youth. We examined the demographic and forensic risk factors for self-harm in youth in juvenile detention using a Canadian provincial correctional database.

**Method:**

We analyzed data from de-identified youth aged 12 to 18 at the time of their offense who were in custody in a Manitoba youth correctional facility between January 1, 2005 and December 30, 2010 (N = 5,102). Univariate and multivariate logistic regression analyses determined the association between staff-identified self-harm events in custody and demographic and custodial variables. Time to the event was examined based on the admission date and date of event.

**Results:**

Demographic variables associated with self-harm included female sex, lower educational achievement, older age, and child welfare involvement. Custodial variables associated with self-harm included higher criminal severity profiles, younger age at first incarceration, longer sentence length, disruptive institutional behavior, and a history of attempting escape. Youth identified at entry as being at risk for suicide were more likely to self-harm. Events tended to occur earlier in the custodial admission.

**Interpretation:**

Self-harm events tended to occur within the first 3 months of an admission stay. Youth with more serious offenses and disruptive behaviors were more likely to self-harm. Individuals with problematic custodial profiles were more likely to self-harm. Suicide screening identified youth at risk for self-harm. Strategies to identify and help youth at risk are needed.

## Introduction

Admission into a correctional facility is a highly stressful event, which can precipitate suicidal ideation, suicide attempts, and self-harm among at-risk youth. Incarcerated youth have greater rates of psychiatric illnesses than age-matched peers, and those with mental disorders tend to be detained longer [1]. Suicide is the number one cause of death among incarcerated youth [2–5].

Previously published studies have a number of limitations that restrict our knowledge of suicide and self-harm among incarcerated youth. Studies examining suicide attempts in young detainees, have relied exclusively on self-report [6–8], which has been problematic due to recall bias and inconsistent reporting on retesting [9](Putnins, 2005). Institutional data [2], [5], [10] has not measured characteristics beyond race, age, and gender and has not determined other risk factors for this phenomenon. No study to date has examined the report of suicide attempts in detainees by corrections staff, nor has any study examined the relationship between self-report of intent and acts confirmed by staff. Moreover, high-risk periods for incarcerated youth have not been identified. To address the limitations in the previous literature, we aimed to examine demographic and custodial correlates of self-harm using a provincial correctional database. We also assessed periods of risk when self-harm events occurred in custody. The term “self-harm” has been used here to refer to acts of self-injury with or without suicidal intent, which has been commonly used in the past [11]. More recently other terms have been developed, including deliberate self-harm, self-injury, and non-suicidal self-injury. The difference in nomenclature and lack of standardized assessments limit our knowledge of this phenomenon [12]; however, it is clear that this behavior is a poor prognostic indicator of both poor physical health and decreased life expectancy [13].

## Materials and Methods

Data were obtained from the Manitoba Government Department of Justice Corrections Information System database in Canada. Participants were de-identified youth aged 12 to 18 years at the time of their offense who were in custody sometime between January 1, 2005 and December 31, 2010 (*N* = 5,102). No participants were excluded from the study. The project was granted judicial approval prior to its commencement and ethics approval was also granted from the Human Research Ethics Board at the University of Manitoba.

### Measures

#### Suicide attempts while in custody

A search for keywords within the database of records of daily events and incident reports was conducted including the terms “suicide”, “self-harm”, and “self-injury”. If any of those keywords appeared in the record, the individual was coded as having self-harmed in the past year. The first incident of self-harm within the timeframe was used for each participant.

#### Sociodemographics

Characteristics including sex, age, level of education, language, and religious affiliation were reviewed. Age (12 to 15 years, 16 years, 17 years, and 18 years and older) and educational level (no education to grade 8, grade 9–10, grade 11 and higher) were categorized based on the distribution of the data. We distinguished English-only speakers from those listing another primary language. Religious affiliation was dichotomized into having a religious affiliation and no affiliation. Child welfare involvement (CWI) was defined as the presence of family supports and/or home placements with social workers.

#### Suicide and security risk factors

The Inmate Security Assessment (ISA) obtained data on every youth within 24 hours of each detention entry to assess risk of suicide and harm to others [14]. Cut-off norms for the risk categories were previously established through a test study of 1000 assessments (e.g., the cut-off level for very high should ensure that no more than 5 percent of the population receive this score) [14]. Data was entered by front line unit staff working directly with youth.

The ISA contained two main sections: suicide and security risk. The suicide risk assessment listed 3 primary indicators of suicide risk, including current suicide plan, feeling alone, and prior suicide history. This latter indicator included both knowledge of a suicide by another person as well as the detainee reporting a suicide attempt in the past. Two secondary indicators included feelings of loss/stress and hopelessness/helplessness. Youth were labeled as showing “no evidence” of suicide if primary and secondary indicators of suicide were negative. A “low” risk of suicide was established if there was a prior suicide attempt or if both secondary indicators were present. A “medium” suicide risk was discerned if there was no clear plan for suicide but suicidal ideation was present or if there was a previous history of self-harm behavior, youth believed they were alone or lacked resources, and there was evidence of one or more secondary indicators. A “high” suicide risk rating was conferred if youth stated a clear and current plan to attempt suicide or youth had a plan combined with one or more primary or secondary indicators of suicide.

The security risk assessment included eight indicators of security risk, including severity of the most serious current offense; severity of the most serious past conviction; number of past convictions; age at first incarceration; custodial sentence length; number of past custody commitals; past institutional behavior; and history of escape, being unlawfully at large, or having a parole revocation in the past 3 years. Each indicator was scored on a 4-point scale. Scores of 0 to 3 were given for each category, with a score of 3 being given for more serious behavior. Total scores were summed across the eight categories. A “low” offense risk was given if the total score was under 2, while “medium” and “high” risk ratings were given if the total score was between 3 and 8, or above 9, respectively.

#### Statistical analyses

First, we used cross-tabulations to examine the prevalence of self-harm across all of the included correlates. Second, using univariate logistic regression analyses, we explored the relationship between sociodemographic correlates and self-harm. Third, we analyzed the relationship between correlates as measured on the suicide and security assessments in relation to self-harm while in custody. Using univariate and multivariate logistic regression, we adjusted first for sociodemographic variables, and second for both sociodemographic variables as well as CWI. Finally, time to the event was examined through chi-square analysis based on the admission date and date of event.

## Results

Table 1 lists the demographic correlates of detained youth who self-harmed. During the study period, 3.5% (*n* = 128) of boys and 8.8% (*n* = 113) of girls self-harmed. Events were associated with female sex (Odds Ratio (OR) 2.5, 95% Confidence Interval (CI) 1.9 to 3.3), having less than grade 11 education (OR 2.2, 95% CI 1.4 to 3.6), and having CWI (OR 5.7, 95% CI 4.4 to 7.6). Younger teens, aged 12 to 15 years, were less likely to self-harm (OR 0.5, 95% CI 0.3 to 0.9). There was no association between self-harm and either language or religion.

**Table 1:** Demographic correlates of self-harm while in youth custody during most recent incarceration.

	No SH (n = 4896)		SH (n = 241)		OR	95%CI
	n	%	n	%		
**Sex**						
Male	3622	74.0	128	53.1	1.0	
Female	1271	26.0	113	46.9	2.5**	1.9, 3.3
**Age (at intake/assessment)**						
12 to 15 years	770	18.3	33	14.0	0.5**	0.3, 0.9
16 years	827	19.7	44	18.7	0.7	0.4, 1.0
17 years	2052	48.8	114	48.5	0.7	0.5, 1.0
18 years and older	557	13.2	44	18.7	1.0	
**Current educational attainment**						
No education to grade 8	1179	25.5	69	29.1	2.2**	1.4, 3.6
Grade 9–10	2566	55.4	145	61.2	2.2**	1.4, 3.4
Grade 11 to some post-secondary	884	19.1	23	9.7	1.0	
**Language spoken**						
English only	4674	95.8	232	96.3	1.0	
Bilingual or other language	203	4.2	9	4.2	0.9	0.5, 1.8
**Religious affiliation**						
Any religious affiliation	1228	25.1	56	23.2	1.0	
No religious affiliation	3668	74.9	185	76.8	1.1	0.8, 1.5
**Child and Family Services involvement**						
No	3681	75.2	83	34.4	1.0	
Yes	1215	24.8	158	65.6	5.7**	4.4, 7.6

**p < .01.

SH = self-harm; OR = odds ratio; 95% CI = 95% confidence interval.

Tables 2 and 3 detail the offense- and suicide-related correlates of self-harm in custody. Even after adjusting for demographics and CWI, self-harm were significantly and positively associated with a number of offense-related factors. Individuals with “medium” or “high” current or past offense severity ratings had significantly increased odds of self-harm (AOR 4.0, 95% CI 2.8 to 5.6 for “high” past offense). Having more than 2 previous convictions was also significantly and positively associated with self-harm(OR 4.8, 95% CI 3.5 to 6.6 for more than 5 convictions). Events were also significantly and positively associated with a younger age at first incarceration (OR 2.5, 95% CI 1.8 to 3.6 for incarceration before age 14), longer custodial sentences (OR 3.0, 95% CI 1.5 to 5.9), multiple incarceration periods (OR 3.5, 95% CI 2.5 to 4.7 for more than 2 incarcerations), previous negative institutional behavior (OR 11.1, 95% CI 8.0 to 15.2 for more than 3 disciplinary actions), and elopements in the previous 3 years (OR 5.0, 95% CI 3.2 to 7.9).

**Table 2:** Offense-related correlates of self-harm during most recent incarceration.

	AOR-1	95%CI	AOR-2	95%CI
**Offense severity rating—current**				
Low	1.0		1.0	
Medium	1.4*	1.0, 2.0	1.5*	1.0, 2.0
High	2.1**	1.5, 2.9	2.4**	1.7, 3.3
**Offense severity rating—most serious past**				
Low	1.0		1.0	
Medium	2.4**	1.7, 3.4	1.9**	1.3, 2.7
High	5.1**	3.6, 7.1	4.0**	2.8, 5.6
**Number of past convictions**				
2 or less	1.0		1.0	
3 to 5	2.1**	1.3, 3.5	1.7*	1.0, 2.8
More than 5	5.0**	3.6, 7.0	3.6**	2.5, 5.1
**Age at first incarceration**				
14 years or younger	3.0**	2.1, 4.3	2.6**	1.8, 3.8
15 to 17 years	2.2**	1.6, 3.1	2.3**	1.7, 3.3
18 years or older	1.0		1.0	
**Custody sentence length**				
Remand or less than 12 mo	1.0		1.0	
12 to 18 months	1.5	0.6, 3.9	1.5	0.6, 3.9
Over 18 months	3.3**	1.6, 6.8	3.6**	1.7, 7.5
**Number of past youth custody commitals**				
None	1.0		1.0	
One	2.4**	1.6, 3.6	2.0**	1.4, 3.0
Two or more	4.2**	3.0, 5.9	3.2**	2.3, 4.6
**Past institutional behavior (last 3 years)**				
No or minor problems	1.0		1.0	
1 or 2 disciplinary boards	5.0**	3.2, 8.0	4.2**	2.7, 6.7
3 or more disciplinary boards	18.6**	12.9, 27.0	13.9**	9.5, 20.3
**History of escape, UALs, parole revocations (last 3 years)**				
None	1.0		1.0	
At least 1 in last 3 years	4.3**	2.6, 7.0	3.7**	2.3, 6.1
At least 1 in last year	3.7**	2.7, 5.1	2.9**	2.1, 4.0

*p < .05.

**p < .01.

UAL = unlawfully at large. AOR-1 = adjusted odds ratio; adjusted for sex, age (continuous), educational level, language spoken, and religious affiliation. AOR-2 = adjusted odds ratio; adjusted for sex, age (continuous), educational level, language spoken, religious affiliation, and child welfare involvement. 95%CI = 95% confidence interval.

**Table 3:** Suicide-related correlates of self-harm during most recent incarceration.

	AOR-1	95%CI	AOR-2	95%CI
**SH while detained, prior to current incarceration**				
No	1.0		1.0	
Yes	5.0**	2.2, 11.7	3.9**	1.6, 9.2
**Assessed suicide risk level at intake**				
No evidence	1.0		1.0	
Low	11.3**	6.5, 19.7	9.3**	5.3, 16.2
Medium	17.8**	9.3, 34.0	14.9**	7.8, 28.7
High	39.5**	14.8, 105.3	31.8**	11.7, 86.8
**History of suicide (own or significant other)**				
No	1.0		1.0	
Yes	14.0**	7.6, 25.8	11.3**	6.1, 20.9
**Suicide plan reported at intake**				
No	1.0		1.0	
Yes	12.0**	5.3, 27.2	7.4**	3.2, 17.1
**Suicidal thoughts reported at intake**				
No	1.0		1.0	
Yes	3.5**	2.1, 5.7	2.4**	1.5, 4.1

**p < .01.

SH = deliberate self-harm. AOR-1 = adjusted odds ratio; adjusted for sex, age (continuous), educational level, language spoken, and religious affiliation. AOR-2 = adjusted odds ratio; adjusted for sex, age (continuous), educational level, language spoken, religious affiliation, and child welfare involvement. 95% CI = 95% confidence interval.

With regard to suicide-related correlates, a history of previous self-harm was associated with self-harm during incarceration. Specifically, knowledge of another’s suicide or suicidal behavior by the participant, expressed suicidal plans and suicidal thoughts at intake were all significantly and positively associated with self-harm. Identification as being at “high” (OR 37.9, 95% CI 14.9 to 96.0), “medium” (OR 21.3, 95% CI 11.5 to 39.5), or “low” (OR 13.3, 95% CI 7.8 to 22.6) suicide risk was significantly and positively associated with self-harm during the current incarceration period compared to those with “no evidence”.

Table 4 provides information regarding the time from admission before a youth self-harmed. The mean time to the event was 102 days while the median time to the event was 42 days. A chi-square analysis indicated that the distribution was significantly skewed, suggesting that self-harm occur more often in the first 3 months of the incarceration period (p < .001).

**Table 4:** Time to self-harm event after admission during most recent incarceration.

	n	%
**Time to SH after admission**		
Within first 30 days	101	42.6
31–90 days	67	28.3
91–365 days	57	24.1
After 1 year	12	5.1

SH = self-harm

## Discussion

This is the first study to our knowledge that examines risk factors for self-harm in incarcerated youth using information from front-line staff. These are important data as they include first-hand information not subject to self-report recall bias. Youth were more likely to self-harm in the first 90 days in custody. Girls, older adolescents, those with less education, and those with CWI were more likely to self-harm. Detainees with aggressive criminal histories as well as those who had a history of previous self-harm were more likely to self-harm in custody.

We found that youth who self-harmed were more likely to to do so in the first 90 days in custody. This is consistent with literature focused on adults that identified the pre-trial period [15] and the time immediately upon incarceration [16] to be high-risk periods for suicide. In contrast, Hayes [17] did not find an elevated risk period for deaths by suicide in his survey of juvenile correctional facilities. That study differed its examination of deaths by suicide, rather than self-harm. Our study highlights the importance of universal screening upon entry and careful monitoring in the earlier part of the detention period in order to identify and help those who are struggling with suicidal thoughts and plans.

Our study demonstrated differences in self-harm risk profiles for incarcerated youth. With regard to demographic variables, detained adolescent girls were more likely to self-harm, which is consistent with previous findings among adolescents in the community [18], [19]. Younger youth were less likely to self-harm, which has also been noted previously among non-incarcerated youth [20]. Shaffer and Fisher [21] hypothesized that suicide attempts increase post-puberty due to the pubertal onset of depression and substance abuse. Consistent with other reports in the general population [22], [23], youth with less education were more likely to self-harm. Individuals who had CWI were at increased risk to self-harm. This is consistent with previous studies looking at non-detained youth [24], [25], though CWI has been associated with decreased suicide attempts following its initiation [26].

The youth who had the most serious convictions, were incarcerated at a younger age, attempted an escape from their legal obligations, and had more aggressive behavior while in custody were more likely to self-harm. This concurs with previous studies in the adult forensic population [27–30] and adolescents in the community [31–33]. Abnormalities of serotonin and noradrenergic functioning have been implicated in both aggressive impulsivity and suicidal behavior [34], [35].

With regard to the suicide risk assessment, youth who had previously self-harmed were more likely to self-harm again while in custody. This is consistent with previous research in the community population, which has identified the repetitive nature of suicidality [30]. Individuals who were identified at “high” risk for suicide were most likely to self-harm, though youth rated at “medium” or “low” risk also self-harmed more frequently compared to youth with “no evidence”. Perhaps those individuals who were at “low” or “medium” suicide risk were able to self-harm due to the lower precautions taken by staff. In addition, the desire to attempt suicide or self-harm can fluctuate over time, and youth who were rated at “low” risk on intake may have experienced a stressor that increased their risk of self-harming while in custody. Knowledge about the suicide of another person or a previous suicide attempt was also associated with an increased rate of self-harm. This could be due to a contagion effect whereby suicidal behavior in one person could lead to similar behavior among peers in their social networks [36]. Youth who expressed either suicidal thoughts or a suicide plan were at greater risk of self-harm. This finding is consistent with hospital [37] and community [30] populations.

Several limitations exist in the present study. First, although we had information about suicide intent at the time of admission into detention, we were unable to discern the intent behind self-harm at the time of the event. Adult forensic research has attempted such a distinction [38]. Second, mental disorder diagnoses and history were not available. Third, the intake question about prior suicide history did not separate previous suicide attempts by the participant from knowledge of suicide in others. Fourth, repeated self-harm in custody was not examined. Fifth, only events documented by staff were included in the study. If events occurred that were not noted by staff, were not entered appropriately into the computer system, or deemed inconsequential, data would be missing. It is also possible that recall bias could have played a role in the identification of the event. Despite these limitations, our study found important information about the characteristics of juvenile detainees who self-harmed in custody.

## Conclusions

Our study demonstrated that youth with the most serious criminal profiles and who were identified as having some suicide risk were most likely to self-harm. The first 3 months of incarceration were a higher risk time period for self-harm. Not only do clinicians need to be especially vigilant in providing support to youth at the earlier parts of admission, additional supports are needed as this is a particularly stressful experience for youth. Future studies should include an examination of the methods used to self-harm and intent to die; and additional research is needed in order to distinguish the characteristics between youth who are classified as being at “high”, “medium” and “low” suicide risk. There may be a need to adopt another mechanism to identify youth at risk of suicide. Once high-risk youth are identified, intervention that occurs both in detention as well as post-custody will hopefully decrease rates of suicide attempts and self-harm in this vulnerable population. Incarceration can be viewed not only as a punitive process, but also an opportunity to engage resources that can help youth re-integrate successfully into community life.
